# Effective prediction of postoperative complications for patients after open hepatectomy: a simplified scoring system based on perioperative parameters

**DOI:** 10.1186/s12893-019-0597-2

**Published:** 2019-09-05

**Authors:** Long Chen, Yun-Bing Wang, Yan-Hong Zhang, Jun-Fei Gong, Yue Li

**Affiliations:** 1 Department of Hepatobiliary Surgery, The Second Affiliated Hospital of Chongqing Medical University, No. 76, Linjiang Road, Yuzhong District, Chongqing, 400010 China; 2 Department of Gastroenterology, The Second Affiliated Hospital of Chongqing Medical University, Chongqing, 400010 China

**Keywords:** Hepatectomy, Complication, Prediction, Risk factor, Scoring system

## Abstract

**Background:**

The aim of the study was to develop a scoring system for the prediction of postoperative complications of open hepatectomy.

**Method:**

All consecutive patients receiving open hepatectomy from 2015 to 2017 were included in the study. Univariate and multivariate analyses were used to confirm the risk factors for postoperative complications. Afterwards, a novel scoring system was developed to predict the postoperative complications.

**Results:**

The study included a total of 207 patients. For the test dataset, multivariate analysis indicated that diabetes, scale of surgery, serum potassium, and blood loss versus body weight were independent risk factors of the postoperative complications. The area under the curve (AUC) of the novel scoring system we proposed for prediction of postoperative complications of hepatectomy was 0.803, which is comparable with the AUCs of previous scoring systems. Furthermore, in the validation dataset, the corresponding AUC of the new scoring system was 0.717.

**Conclusion:**

This novel and simplified scoring system can effectively predict the postoperative complications of open hepatectomy and could help identify patients who are at high risk of postoperative complications.

**Electronic supplementary material:**

The online version of this article (10.1186/s12893-019-0597-2) contains supplementary material, which is available to authorized users.

## Introduction

Hepatectomy is a curative method for hepatocellular carcinoma (HCC) according to the Barcelona Clinic Liver Cancer (BCLC) staging system [1, 2]. In recent years, indications for hepatectomy have been broadened (not only for malignant liver disease but also for benign liver disease) because of the improved safety of liver resection [3, 4]. In the last century, mortality from hepatectomy was approximately 9–20% or more [5–7]. Thanks to the high-speed development of surgical techniques and advances in perioperative healthcare, mortality after liver resection has decreased to approximately 3–10% and is even lower in different volume centers [8–10]. In contrast, the complication rate after open hepatectomy remains to be high, ranges from 20 to 48% and shows no tendency to decline [11–13]. The occurrence of postoperative complications not only increases the difficulty of postoperative healthcare but also prolongs the hospital stay and leads to high costs in the hospital. A validated and reliable scoring system to predict postoperative complications of hepatectomy is indispensable for prediction of postoperative complications and doctor-patient communication. Therefore, more and more studies have been focused on the strategies not only to prevent the mortality but also to predict the nonlethal postoperative complications. Many studies have been proposed to identify risk factors of postoperative complications and then to help patients not at high risk of poor outcome after surgery [14–16].

Existing scoring systems, such as Physiologic and Operative Severity Score for the enUmeration of Mortality and Morbidity (POSSUM) [17], Estimation of Physiologic Ability and Surgical Stress (E-PASS) [18], Acute Physiology and Chronic Health Evaluation II (APACHE-II) [19] and APACHE-III [20] were developed to predict the mortality or morbidity of patients after surgery, but all of them showed no convenience to predict the risk of postoperative complications, due to the variety of parameters and complex formulas. The “50–50 criteria” developed by Balzan S et al. [21] is a model to identify potential complications, which indicates that prothrombin time < 50% and serum bilirubin > 50 μmol/L on postoperative day 5 is a simple and accurate predictor for mortality and morbidity after hepatectomy. However, this method has a limitation in that it is constructed on the basis of the postoperative parameters that are available on postoperative day 5 after the liver resection. This limitation leads to its uselessness to help surgeons to predict the occurrence of postoperative complications as soon as possible.

In this study, to develop a widely accepted and reliable scoring system, we gathered together all the parameters that were included in POSSUM, E-PASS, APACHE-II and APACHE-III, and we then added some factors associated with liver diseases. By analyzing the post-hepatectomy complications based on the Clavien-Dindo staging system [22, 23], we developed a new, simplified and reliable scoring system for prediction of postoperative complications.

## Methods

### Patients and study design

The study accords with the ethics of the Second Affiliated Hospital of Chongqing Medical University. Data from 207 consecutive patients who suffered from benign or malignant hepatobiliary disease and underwent traditional open hepatectomy in the Department of Hepatobiliary Surgery, the Second Affiliated Hospital of Chongqing Medical University from 2015 to 2017 were collected retrospectively. Benign hepatobiliary disease included hepatolithiasis, hemangioma, focal nodular hyperplasia, adenoma, liver cyst, choledochal cyst, liver abscess and so on. Malignant hepatobiliary disease mainly included primary HCC and intrahepatic cholangiocarcinoma.

The indications for surgery have been described in detail by previous studies [3, 7]. In this study, all cases of the disease were diagnosed by ultrasonography combined with contrast computed tomography (CT) scan or magnetic resonance imaging (MRI). Before surgery, all patients’ liver function was assessed according to the Child-Pugh classification, and the postoperative liver remnant was also assessed by CT volumetry. Patients with Child-Pugh A or Child-Pugh B were fit for hepatectomy [24]. Operations were performed by all surgical teams in our department. All the operations were started with conventional inverted L incision, and the change of incision depended on the intraoperative conditions, such as tumor location or the necessity of surgical field. When necessary, Cavitron ultrasonic aspirator (CUSA) was used as an assistant tool for liver resection. Postoperative drainage tube was routinely placed. Normally, patients were allowed to drink a small amount of water on postoperative day 1, liquid diet on day 2 and normal diet from day 3 to the date of discharge. The postoperative complications were collected from the Electronic Medical Record System, and the primary outcome was postoperative complication. To objectively evaluate the postoperative complication, it was graded according to Clavien-Dindo complications classification [22, 23]. This study was supposed to use our newly proposed scoring system to answer the question whether the patients would suffer the postoperative complication. So, the postoperative complication in this study represents one type of dichotomous variable.

### Selection of scoring systems and predictive variables

POSSUM [17], E-PASS [18], APACHE-II [19] and APACHE-III [20] were gathered for the evaluation of the predictive ability of postoperative complications. Physiological score (PS) and operative score (OS) make up the POSSUM scoring system. Twelve variables were included in PS, and six variables were included in OS. Each variable has its value and corresponding score. Therefore, PS, OS, and total score (TS; TS=PS + OS) can be calculated. E-PASS is made up of six preoperative risk factors and three operation-associated risk factors. In addition, the preoperative risk score (PRS), the surgical stress score (SSS), and the comprehensive risk score (CRS) could be calculated. The three components of APACHE-II are acute physiological score (APS), chronic health status (CHS), and patient’s age. APACHE-II has been widely used in clinical practice due to its dependability and convenience and the higher the score is, the higher the mortality and poorer prognosis of the patient. APACHE-III is made up of APS, CHS, and patient’s age, which is similar to the components of APACHE-II. APACHE-III includes several new variables, and the scores of the old variables are also optimized.

In the study, the variables in these classic scoring systems were considered to be potential variables included in our newly proposed scoring system. Additionally, we added some variables that were not presented in those previous scoring systems but are closely associated with liver diseases, such as HBV infection, HCV infection, aspartate aminotransferase (AST), alanine aminotransferase (ALT), alkaline phosphatase (ALP), gamma-glutamyl transpeptidase (GGT), direct bilirubin (DBIL), indirect bilirubin (IBIL), prothrombin activity (PTA), prothrombin time (PT), activated partial thromboplastin time (APTT), thrombin time (TT), and serum alpha-fetoprotein (AFP).

### Statistical analysis

In this study, we used SPSS 23.0 software for the analysis. Before data analysis, patients were randomly allocated into the test group and the validation group with the method of random number table (seed: 20180317). Continuous variables are expressed as the mean ± standard deviation (SD) and were compared with independent sample t-test between groups. Categorical variables are displayed as the number and corresponding percentage and compared with Chi-square test between groups. The postoperative complication was objectively evaluated with the Clavien-Dindo staging system, as described above. In the test group, each variable was assigned with a score according to the previous scoring system, the receiver operating characteristic (ROC) curve was graphed, and the area under the curve (AUC) was calculated to evaluate whether those previous scoring systems could accurately predict the occurrence of the postoperative complication for patients after open hepatectomy.

Univariate analysis (with logistic regression) was performed to identify the variables that were associated with postoperative complications of hepatectomy. Risk factors were identified after the univariate analysis. Next, multivariate analysis with logistic regression and forward likelihood ratio test was performed to determine independent risk factors that were closely associated with the incidence of postoperative complications. All the variables found to be significant in univariate analysis were included into the multivariate analysis. Afterwards, the predicted probability for the postoperative complication was used for the evaluation of predictive ability. After that, the ROC curve was graphed with the predicted values (probabilities), and the efficacy or discrimination of the model with several variables combined against the incidence of postoperative complication was assessed by the AUC. The calibration of the model was evaluated by Hosmer-Lemeshow good of fit test, and meanwhile the calibration plot was graphed to visualize the result of calibration. Additionally, each independent risk factor was assigned with a score of zero or one based on the size of the odds ratio (OR), and a new total score was obtained for each patient. After that, according to the total score, and the efficacy of the new scoring system against the incidence of postoperative complication was assessed again by ROC curve and AUC. Additionally, Chi-square test was used to analyze the relationships between the occurrence of postoperative complications and the obtained total score. Finally, the efficacy of the new scoring system for predicting the incidence of postoperative complication was validated in the validation group through ROC and AUC value. *P* < 0.05 was considered to be significant.

## Results

### Patient characteristics

From 2015 to 2017, there were 207 consecutive patients (135 males and 72 female) who underwent traditional open hepatectomy in the Second Affiliated Hospital of Chongqing Medical University for both benign and malignant liver disease. After randomization, the cohort of 207 patients was stratified into two groups: 150 patients in the test group, and 57 patients in the validation group. We found that 68.667% of the patients in the test group were male, and 56.140% of the patients in the validation group were male. The mean age was 53.89 ± 12.38 years and 54.32 ± 11.29 years in the test and validation groups, respectively. Hepatic cirrhosis accounted for 42.667% in the test group and 40.351% in the validation group. Furthermore, 8.000% of the patients in the test group and 8.772% in the validation group had a disease history of diabetes. Additionally, 64.000% of the patients in the test group and 56.140% of the patients in the validation group had malignant tumor, which had been confirmed by pathology. No difference was found between the test and validation groups in gender, age, history of serious lung disease, history of serious heart diseases, HBV infection, HCV infection, hepatic cirrhosis, diabetes, existence of tumor, operative time, or length of hospital stay (all with P > 0.05; Table 1). In the whole study, the overall complication rate was 18.4%. The condition of postoperative complications was evaluated with Clavien-Dindo staging system in detail (Table 2).

**Table 1:** General traits of the patients in test group and validation group

Variables	Categories	Test set	Validation set	Total	*P* value
Gender	Male	103	32	135	0.104
	Female	47	25	72	
Age		53.89 ± 12.38	54.32 ± 11.29		0.823
History of serious lung diseases	No	147	57	204	0.563
	Yes	3	0	3	
History of serious heart diseases	No	143	54	197	1.000
	Yes	7	3	10	
HBV	No	86	33	119	1.000
	Yes	64	24	88	
HCV	No	148	57	205	1.000
	Yes	2	0	2	
Hepatic cirrhosis	No	86	34	120	0.875
	Yes	64	23	87	
Diabetes	No	138	52	190	0.785
	Yes	12	5	17	
Tumor	No	54	25	79	0.338
	Yes	96	32	128	
Operative time (minutes)		239.94 ± 100.60	250.70 ± 114.00		0.517
Length of hospital (days)		19.79 ± 9.32	21.95 ± 11.13		0.160

Note: *HBV* = hepatitis B virus; *HCV* = hepatitis C virus

**Table 2:** Dindo-Clavien staging system for evaluation of postoperative complication

Variables	Categories	Test set	Validation set	Total	Percentage
Complication (Yes or No)	No	124	45	169	81.6%
	Yes	26	12	38	18.4%
Complication (Dindo stage)	0	124	45	169	81.6%
	2	6	3	9	4.3%
	3a	16	7	23	11.1%
	3b	0	1	1	0.5%
	4a	2	1	3	1.5%
	4b	1	0	1	0.5%
	5	1	0	1	0.5%

### Development of the new scoring system

In the test dataset, univariate analysis was used to identify potential risk factors of the complication after open hepatectomy. Afterward, seven risk factors were found to be associated with the postoperative complication (Additional file 1: Table S1), including diabetes [OR: 3.980, 95% confidence interval (CI): 1.154–13.725, P = 0.029], intraperitoneal pollution (OR: 1.441, 95% CI: 1.012–2.052, P = 0.043), high blood-loss volume (OR: 1.002, 95% CI: 1.001–1.003), high blood loss versus body weight (OR: 1.128, 95% CI: 1.051–1.210, P = 0.001), bigger scale of surgery (OR: 1.689, 95% CI: 1.314–2.170, P < 0.001), more times of surgeries in 30 days (OR: 9.603, 95% CI: 2.133–43.232, P = 0.003) and low serum potassium (OR: 0.157, 95% CI: 0.043–0.573, P = 0.005).

**Fig. 1:**
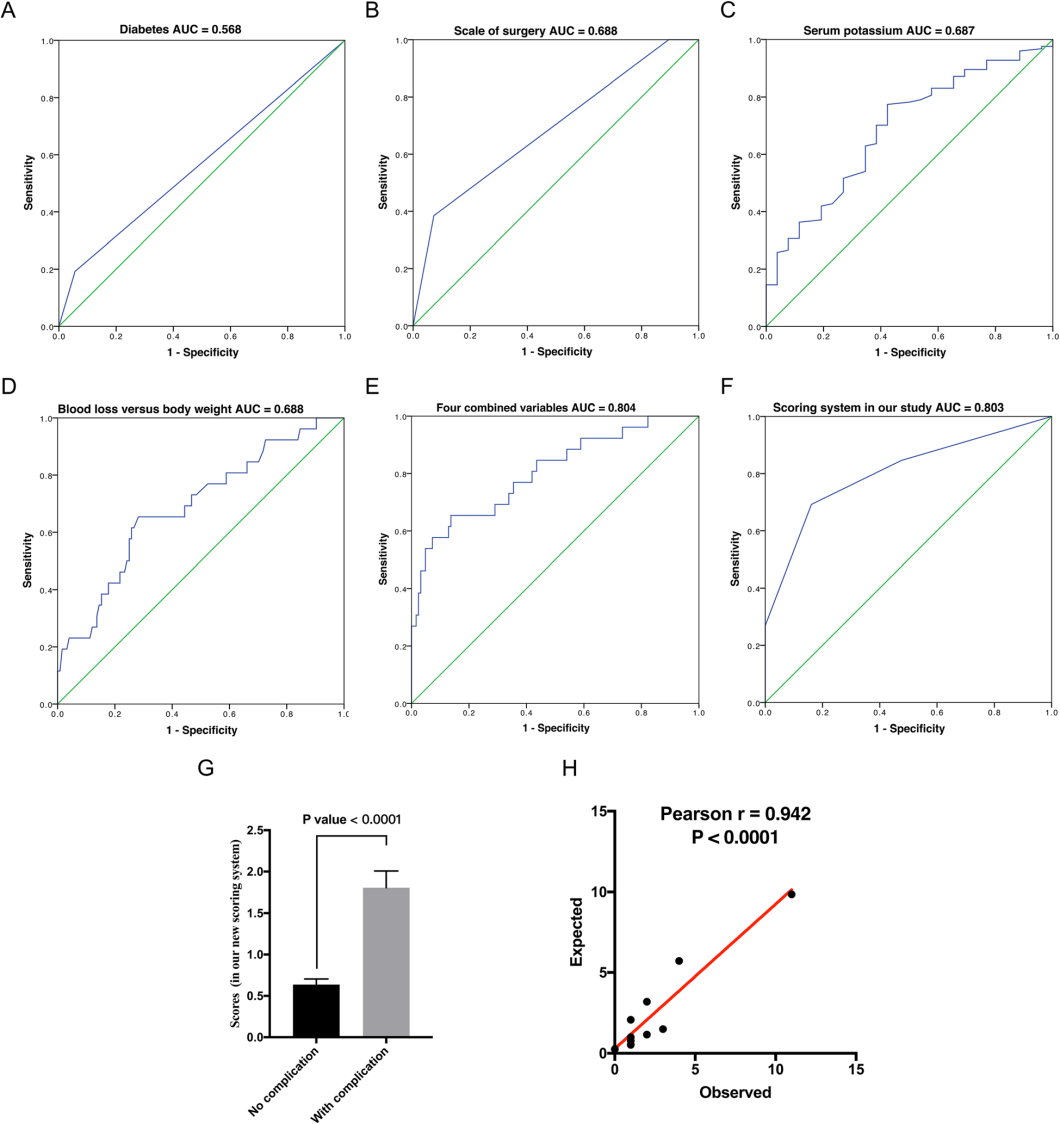
Predictive efficacy of the three variables and the combined variable. Note: With these identified independent risk factors, the ROC curves were independently graphed (**a**, **b**, **c**, and **d**). The AUCs for diagnoses of postoperative complication were 0.568, 0.688, 0.687, and 0.688 for the variables: diabetes, scale of surgery, serum potassium, and blood loss versus body weight, respectively. Furthermore, the AUC of the four combined variables was 0.804 (**e**). After assigning scores 0 or 1 to each variable, the AUC of the newly constructed and simplified scoring system was 0.803 (**f**). In addition, the score of the group with no complications was much lower than that in the group with complications (*P* < 0.0001; **g**). With the multivariate analysis model, Hosmer-Lemeshow test showed the expected value and observed value were positively correlated (Pearson r = 0.942, *P* < 0.0001; **h**); AUC = area under curve; ROC = receiver operating characteristic

Multivariate analysis with forward likelihood ratio selected five variables, which are diabetes, scale of surgery, serum potassium, times of surgery in 30 days, and blood loss versus body weight. Although times of surgery in 30 days was found as an independent risk factor, this parameter might not be a suitable one for risk prediction of postoperative complication as soon as possible. So, in the end, only other four variables were included in the multivariate analysis. The result showed that diabetes (OR: 6.307, 95% CI: 1.464–27.163, P = 0.013), bigger scale of surgery (OR: 1.619, 95% CI: 1.209–2.166, P = 0.001), low serum potassium (OR: 0.167, 95% CI: 0.039–0.703, P = 0.015) and high blood loss versus body weight (OR: 1.079, 95% CI: 1.001–1.163, P = 0.048) were independent risk factors of postoperative complications (Table 3). With these identified independent risk factors, the ROC was graphed independently for each (Fig. 1a, b, c, d), and the novel scoring system was proposed (Table 4). The AUCs for diagnoses of postoperative complication were 0.568, 0.688, 0.687 and 0.688 for variables diabetes, scale of surgery, serum potassium, times of surgery in 30 days and blood loss versus body weight, respectively. In addition, the AUC of the four combined variables was 0.804 (95% CI: 0.704–0.905; Fig. 1e). Furthermore, the cut-off values for diabetes, scale of surgery, serum potassium, and blood loss versus body weight were 0.500, 6.000, 3.835 and 7.846, respectively. When the parameters fit these conditions (≥0.500, ≥6.000, < 3.835 and ≥ 7.846), 1 point was assigned, otherwise 0 point was scored. The predictive effect showed the AUC was 0.803 (95% CI: 0.698–0.909; Fig. 1f), and the score of the group with no complications was much lower than the group with complications (Fig. 1g). Furthermore, with the data in test group, it was found that the incidence of postoperative complication increased with increasing of obtained score (χ^2^ = 48.856, P < 0.001; Additional file 1: Table S2). As we found, the cut-off value for the newly proposed scoring system was 1.500. With this threshold, the incidence of postoperative complication between group with 0–1 points and 2–3 points was significantly different (χ^2^ = 32.041, *P* < 0.001; Additional file 1: Table S2). Additionally, the model presents to be a good calibration, as reflected by the Hosmer-Lemeshow good of fit test (χ^2^ = 5.598, P = 0.692) and calibration plot (Fig. 1h; with a good correlation between expected and observed values; Pearson r = 0.942, *P* < 0.0001).

**Table 3:** Multivariate analysis of the risk factors in test group

Variables	OR (95%CI)	*P* value
Diabetes	6.307 (1.464, 27.163)	**0.013**
Blood loss versus body weight	1.079 (1.001, 1.163)	**0.048**
Scale of surgery	1.619 (1.209, 2.166)	**0.001**
Serum potassium	0.167 (0.039, 0.703)	**0.015**

Note: OR = odd ratio; CI = confidence interval; Bolded P value means it was lower than 0.05

**Table 4:** New scoring system

Variables	Conditions	Scores
Diabetes	No	0
	Yes	1
Scale of surgery	< 6	0
	≥6	1
Serum potassium	≥3.835	0
	< 3.835	1
Blood loss versus body weight	< 7.846	0
	≥7.846	1

Note: Score 0 would be calculated if no diabetes, scale of surgery (< 6), serum potassium (> 3.835), or blood loss versus body weight (< 7.846). When diabetes (yes), scale of surgery (≥6), serum potassium (≤3.835), Blood loss versus body weight (≥7.846) was fulfilled, each parameter would be scored with 1 point. The total score (TS) was calculated as the sum of the four variables

Additionally, because the variable scale of surgery has a shortage of relative subjectivity, we depicted the various scales of surgery and corresponding score according to the POSSUM scoring system reported by previous study [3, 17] (Additional file 1: Table S3). The scale of surgery was divided into three grades (moderate, major, and major+) according to the operative complexity in POSSUM scoring system [17]. Each grade of hepatectomy referred to the study of Poon RT et al. [3]. However, concomitant extrahepatic procedures were sometimes performed, such as hepaticojejunostomy, bowel resection, and other organ operation. Therefore, moderate hepatectomy combined with hepaticojejunostomy, bowel resection or other organ operations was considered as major hepatectomy. Additionally, the major hepatectomy plus hepaticojejunostomy, bowel resection or other organ operation was thought as major+ hepatectomy.

### Evaluation of previous scoring systems in the test group

**Fig. 2:**
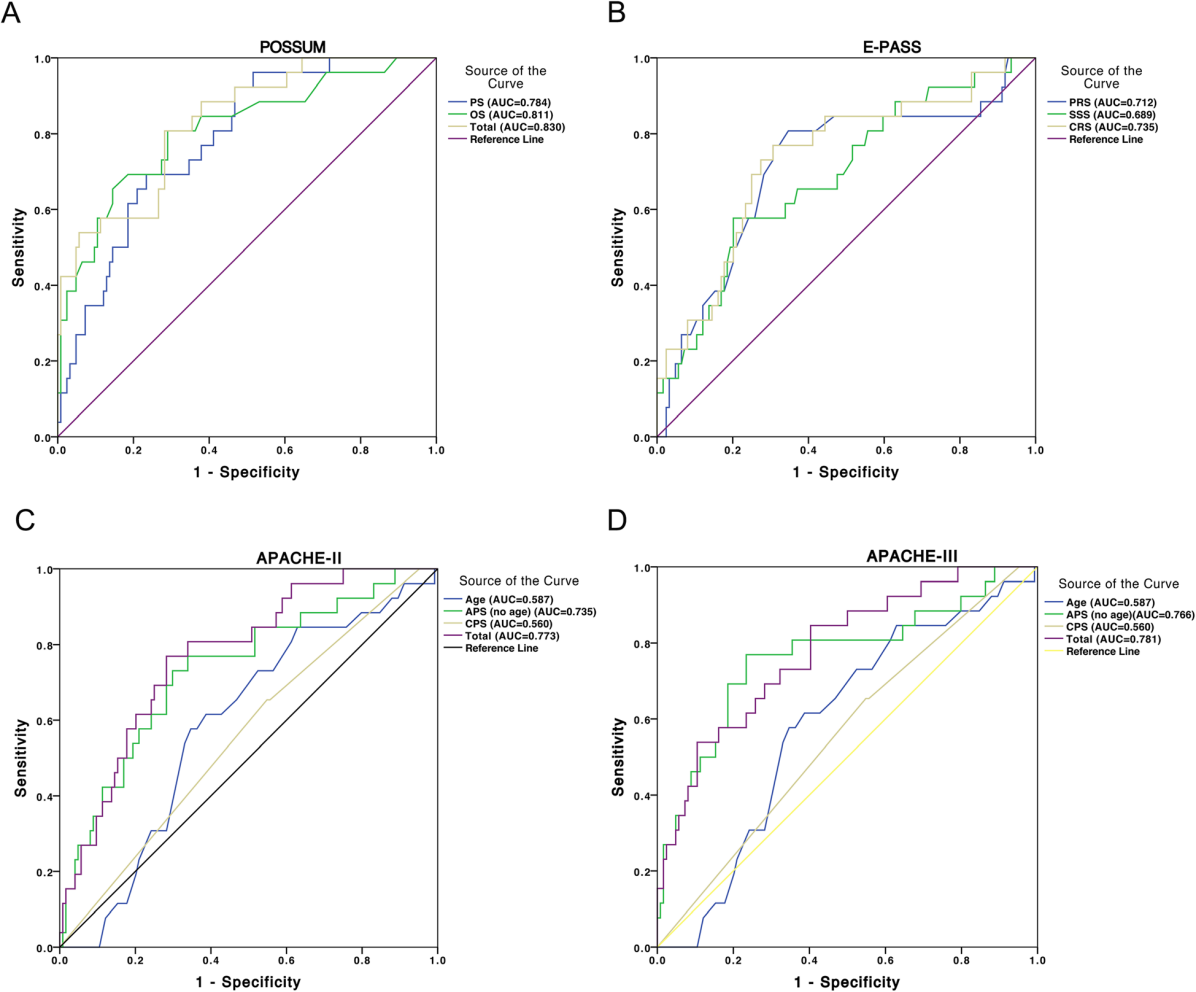
Evaluation of previous scoring systems in the test group. Note: For POSSUM, the AUCs of the PS, OS and TS for the prediction of the postoperative complications were 78.4, 81.1 and 83.0%, respectively (**a**). For E-PASS scoring system, the AUCs of PRS, SSS, and CRS were 71.2, 68.9 and 73.5%, respectively (**b**). The AUCs of APS, age, CPS and TS were 73.5%, 58.7, 56.0 and 77.3%, respectively, in APACHE-II (**c**) and were 76.6, 58.7, 56.0 and 78.1%, respectively, in APACHE-III (**d**). PS = physiological score; OS = operative score; TS = total score; PRS = preoperative risk score, SSS = surgical stress score; CRS = comprehensive risk score; APS = acute physiological score; and CHS = chronic health status

Each patient in the test group was scored according to the parameter and corresponding score in the previous scoring system. For POSSUM, the PS, OS and TS were calculated according to the reported formula [17]. Then ROC of the PS, OS and TS were graphed (Fig. 2a). The AUCs of the PS, OS and TS for the prediction of the postoperative complications were 78.4% (95% CI: 69.8–87.0%), 81.1% (95% CI: 71.3–90.9%) and 83.0% (95% CI: 74.7–91.4%), respectively. For E-PASS scoring system, PRS, SSS, and CRS were calculated, and the corresponding ROCs were graphed for the prediction of postoperative complications of open hepatectomy (Fig. 2b). The AUC of PRS, SSS, and CRS was 71.2% (95% CI: 59.5–83.0%), 68.9% (95% CI: 57.5–80.2%) and 73.5% (95% CI: 62.5–84.5%), respectively. For APACHE-II and APACHE-III, APS, age and CPS were all included in these two scoring systems. TS was equal to the sum of APS, age, and CPS. Next, the ROCs of APS, age, CPS, and TS for prediction of postoperative complication were graphed for APACHE-II (Fig. 2c) and APACHE-III (Fig. 2d). The AUCs of APS, age, CPS and TS were 73.5% (95% CI: 62.7–84.3%), 58.7 (95% CI: 47.6–69.9%), 56.0% (95% CI: 44.3–67.7%) and 77.3% (95% CI: 68.1–86.5%), respectively, in APACHE-II, and 76.6% (95% CI: 65.2–87.9%), 58.7 (95% CI: 47.6–69.9%), 56.0% (95% CI: 44.3–67.7%) and 78.1% (95% CI: 68.5–87.8%), respectively, in APACHE-III.

### Validation of the scoring system

**Fig. 3:**
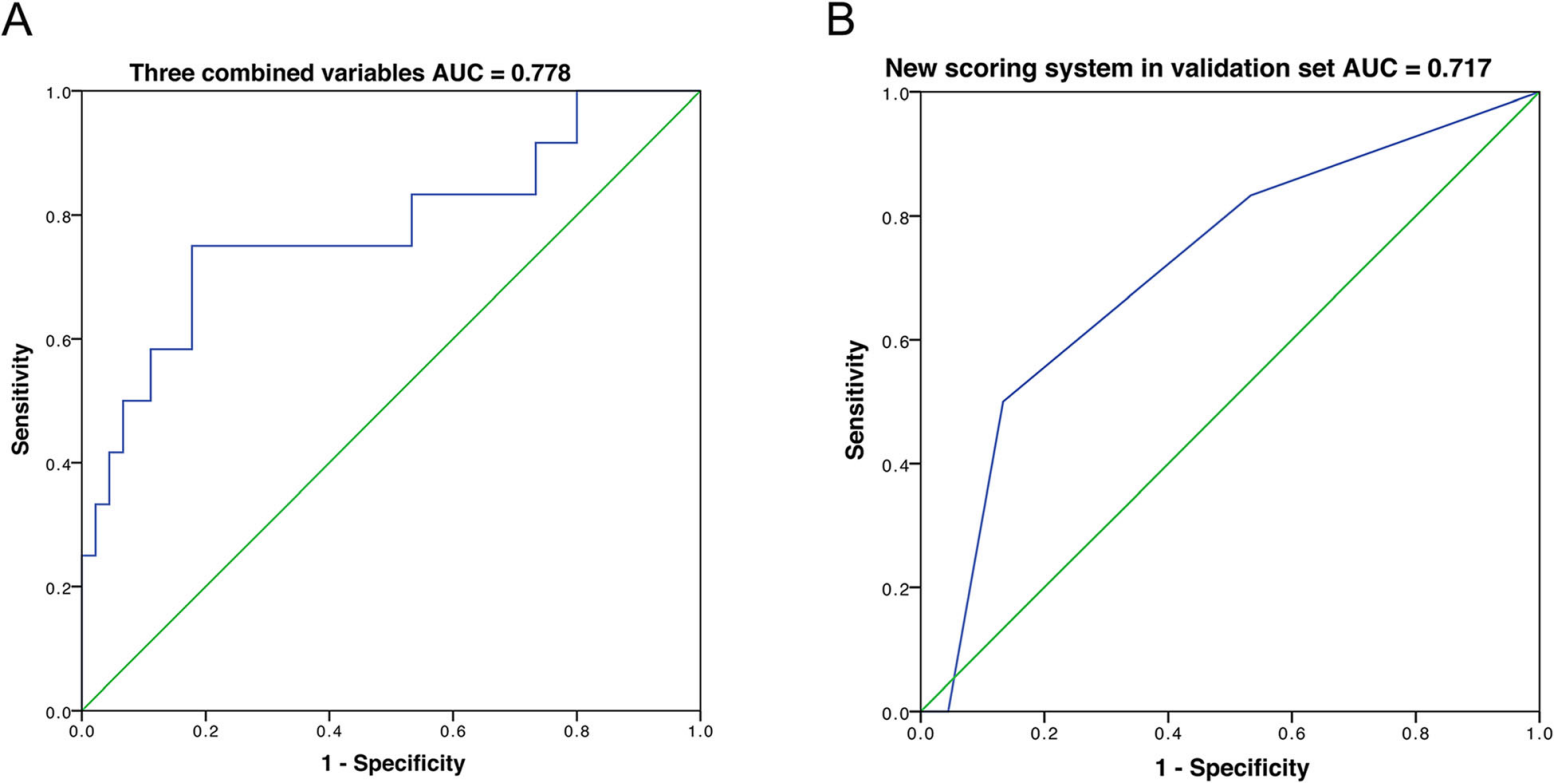
Validation of the new scoring system. Note: With the patients’ data in the validation group, the four independent risk factors found in the test group were combined to predict the postoperative complications, which indicated the importance of these four parameters (AUC = 0.778; **a**). With the planned scoring strategy in Table 4, the new scoring system retained its power in predicting the postoperative complications of open hepatectomy (AUC = 0.717; **b**)

Four independent risk factors in the test group were directly used for the multivariate analysis in the validation group (n = 57). The result indicated the significance of the combination of these parameters for prediction of postoperative complications of open hepatectomy (AUC = 0.778, 95% CI: 0.609–0.947, Fig. 3a). Furthermore, according to the planned scoring strategy in Table 4, the patients in the validation group were scored with the new scoring system. Afterward, another ROC curve was graphed, which indicated that the newly proposed scoring system retained its power in predicting the postoperative complications of open hepatectomy (AUC = 0.717, 95% CI: 0.551–0.883, Fig. 3b).

## Discussion

Based on the univariate and multivariate analyses of the perioperative parameters, we finally found that diabetes, scale of surgery, serum potassium, and blood loss versus body weight were independent risk factors of the postoperative complications of open hepatectomy. The predictive effectiveness of our scoring system was certified in both the test and validation datasets. In this novel and simplified prediction scoring system, patients can get a corresponding risk score according to their perioperative parameters. As we found, the higher score the patients obtained, the higher the postoperative complication rate would be. The novel scoring system was able to assess the risk of postoperative poor outcome. Most importantly, the score can guide the clinical practitioner to make interventions to prevent or to reduce the occurrence of postoperative complications.

In the past, many studies focused on the mortality and long-term survival of the patients after hepatectomy [25–27]. However, limited studies have been developed to determine which perioperative parameters were the risk factors influencing the postoperative complications of liver resection [14–16, 28]. The Surgical Apgar Score (SAS), which was first proposed in 2007 by Gawande et al. [29], is a 10-point model made up of three intraoperative parameters: mean arterial pressure (MAP), heart rate (HR), and estimated blood loss (EBL). Its predictive efficacy of postoperative outcomes has been validated in the past few years in different surgeries, such as gynecological operation [30], gastrointestinal operation [31], and vascular operations [32]. Although SAS was a widely used tool for predicting complications of general or vascular operations, the prediction ability in certain population still need to be increased. Additionally, this tool was not specially designed to certain disease, and the applied value of this tool in liver disease has not been determined.

To our knowledge, a majority of the previous studies were based on malignant liver disease; this study is the first to assess the risk of postoperative complication after hepatectomy for both benign and malignant disease. All the parameters originated from several well-developed scoring systems, which have been widely used in clinical practice to predict the morbidity and mortality after surgery. We made a validation of the predictive ability of these four scoring systems about the complication after hepatectomy, but the process was obviously too complex. In contrast, our newly proposed scoring system and the previous classic scoring system were comparable in the predictive ability for postoperative complication. Importantly, our newly proposed scoring system has been greatly simplified, and the clinical practicality has been enhanced.

Our study identified four independent risk factors of postoperative complication: diabetes, scale of surgery, serum potassium, and blood loss versus body weight. As we know, patients with diabetes have high blood glucose, which is called hyperglycemia. Postoperative hyperglycemia increases the risk of infections in the body and impairs the function of fibroblasts. Traditional open surgery requires a large incision, and the incision healing includes hemostasis, inflammation, proliferation and remodeling. Unfortunately, diabetes can have an impact on all of these processes [33]. Underlying chronic disease, especially for cardiovascular disease and renal failure caused by diabetes, can also increase the risk of incision complications, such as dehiscence and infection. Therefore, diabetes had also been indicated as a risk factor for postoperative complications in several previous studies [34–36].

As the second risk parameter, the scale of surgery was considered to be a procedure-related factor, which can be planned and predicted before the operation. In fact, the open surgery would usually not be changed once the surgical method was determined. The possible reason might originate from the advanced imaging techniques and rigorous preoperative evaluation in our hospital. Compared with other scoring systems, we thought including the scale of surgery is the disadvantage of our proposed scoring system, because this parameter is somewhat subjective. On consideration of this, we tried our best to make the variable (scale of surgery) be described objectively and in detail. Different types of hepatectomy have different wound extents, so postoperative liver remnant was very important to sustain the liver function. When more segments were cut, the risk of liver failure would be increased. Hepaticojejunostomy and bowel resection are the extra procedures for hepatectomy; the reconstruction of anastomosis makes it harder for surgeons to complete the operation, and patients with multiorgan operation are more likely to have complications than single organ operation. That is the reason why we still included the scale of surgery in this scoring system, although it was sometimes thought to be a subjective variable.

Additionally, serum potassium, which had been shown to be a risk factor in elective noncardiac surgery [37] and general thoracic surgery [38], was also considered to be included in the present scoring system. To find what complication low preoperative potassium might lead to, we screen the dataset for the patients with low preoperative potassium. As a result, we found these patients with low serum potassium might suffer incision infection, palmary infection, intra-abdominal infection, intra-abdominal bleeding, and so on. It seems that these complications were not specific. As for the reason, we thought the rationale might be much complicated and need to be explored by mechanism investigation in the future. Finally, the study identified blood loss versus body weight but not blood loss was an independent risk factor of postoperative complication. To reflect the risk of postoperative complication, blood loss corrected by body weight would better than blood loss alone.

In many previous studies, low preoperative serum albumin (hypoalbuminemia) was also reported as a risk factor of postoperative morbidity and mortality [39–42]. In our study, serum albumin was not found to be a risk factor of postoperative complications. The possible reason might be that the serum album level for most patients in our study was in normal range before surgery. In our department, for preoperative preparation, preoperative hypoalbuminemia in certain patients had been always remedied before hepatectomy by the infusion of albumin. Hence, low serum albumin level was not found to be a significant risk factor in our study.

Although this new scoring system is able to predict the postoperative complications of open hepatectomy, there were still some limitations in this study. First, this scoring system is the first to predict the postoperative complications of both benign and malignant liver disease, but it is not clear whether there is a difference between benign and malignant liver disease in the complication risk of hepatectomy. Due to the small sample size, the study did not explore this difference. Therefore, studies with larger sample sizes are expected to be conducted in the future to validate our conclusion. Second, the patients in our study were from a single investigation center. Thus, no validation with patients in multiple research centers is another limitation of our study. Nevertheless, the study had successfully proposed a novel and simplified scoring system, which could effectively predict the perioperative complications of open hepatectomy.

## Conclusions

The novel, validated scoring system has a strong predictive ability for the postoperative complications of open hepatectomy. This system not only allows surgeons to identify patients who are at high risk of postoperative complications but also helps the clinical practitioners undertake better comprehensive preoperative preparations to reduce complications.

## Additional file

Additional file 1: **Table S1.** Univariate analysis of various variables in test group. **Table S2.** Postoperative complication in groups with different score. **Table S3.** Definition for scales of surgery and corresponding scores. (DOC 84 kb)

## Data Availability

The datasets used and/or analysed during the current study are available from the corresponding author on reasonable request.

## Abbreviations

AFP: Alpha-fetoprotein
ALP: Alkaline phosphatase
ALT: Alanine aminotransferase
APACHE: Acute Physiology and Chronic Health Evaluation
APS: Acute physiological score
APTT: Activated partial thromboplastin time
AST: Aspartate aminotransferase
AUC: Area under the curve
BCLC: Barcelona Clinic Liver Cancer
CHS: Chronic health status
CI: Confidence interval
CRS: Comprehensive risk score
CT: Computed tomography
CUSA: Cavitron ultrasonic aspirator
DBIL: Direct bilirubin
EBL: Estimated blood loss
E-PASS: Estimation of Physiologic Ability and Surgical Stress
GGT: Gamma-glutamyl transpeptidase
HCC: Hepatocellular carcinoma
HR: Heart rate
IBIL: Indirect bilirubin
MAP: Mean arterial pressure
MRI: Magnetic resonance imaging
OR: Odds ratio
OS: Operative score
POSSUM: Physiologic and Operative Severity Score for the enUmeration of Mortality and Morbidity
PRS: Preoperative risk score
PS: Physiological score
PT: Prothrombin time
PTA: Prothrombin activity
ROC: Receiver operating characteristic
SAS: Surgical Apgar Score
SD: Standard deviation
SSS: Surgical stress score
TS: Total score
TT: Thrombin time
