# Supplementary material for: Effective prediction of postoperative complications for patients after open hepatectomy: a simplified scoring system based on perioperative parameters
Source: BMC Surg. 2019 Sep 5;19:128. doi: 10.1186/s12893-019-0597-2 (PMC6729098; doi:10.1186/s12893-019-0597-2)
Supplement: Supplementary file 1 — Table S1. Univariate analysis of various variables in test group. Table S2. Postoperative complication in groups with different score. Table S3. Definition for scales of surgery and corresponding scores. (DOC 84 kb) [file 12893_2019_597_MOESM1_ESM.doc]

**Additional file 1**

**Table S1. Univariate analysis of various variables in test** group

| Variables |  | OR (95%CI) | P value |
| --- | --- | --- | --- |
| **Variables presented in previous scoring system** | | | |
| Age |  | 1.017(0.982, 1.054) | 0.340 |
| Chronic health score | | 0.780(0.520, 1.169) | 0.228 |
| Liver cirrhosis |  | 1.187(0.507, 2.775) | 0.693 |
| Diabetes |  | 3.980(1.154, 13.725) | **0.029** |
| Sever heart disease |  | 2.7856E-9(0, NA) | 0.999 |
| Sever lung disease |  | 2.440(0.213, 27.958) | 0.473 |
| Body temperature |  | 2.661(0.579, 12.238) | 0.209 |
| Pulse rate |  | 1.002(0.970, 1.035) | 0.899 |
| Breath rate |  | 0.951(0.813, 1.112) | 0.528 |
| Systolic pressure |  | 0.995(0.964, 1.027) | 0.746 |
| Mean arterial pressure |  | 0.990(0.950, 1.032) | 0.634 |
| Classification of physical state | | 4.920(0.298, 81.310) | 0.266 |
| Emergency operation |  | 1.701(0.668, 4.332) | 0.266 |
| Operative time |  | 1.003(0.999, 1.007) | 0.154 |
| Intraperitoneal pollution | | 1.441(1.012, 2.052) | **0.043** |
| Operative incision size | small | 1.000 |  |
|  | large | 1.651(0.354, 7.704) | 0.523 |
| Blood loss | | 1.002(1.001, 1.003) | **0.001** |
| Blood loss/body weight |  | 1.128(1.051, 1.210) | **0.001** |
| PaO2 |  | 1.002(0.998, 1.006) | 0.401 |
| Scale of surgery |  | 1.689(1.314, 2.170) | **<0.001** |
| Times of surgery in 30 days | | 9.603(2.133, 43.232) | **0.003** |
| ASA stage | low | 1.000 |  |
|  | high | 1.099(0.444, 2.720) | 0.838 |
| Heart symptom |  | 0.299(0.061, 1.453) | 0.134 |
| Respiratory system | | 2.072(0.457, 9.391) | 0.345 |
| Artery blood PH |  | 0.291(0.210, 1.477) | 0.713 |
| PaCO2 |  | 1.044(0.978, 1.114) | 0.200 |
| Hb |  | 0.992(0.970, 1.014) | 0.450 |
| HCT |  | 0.989(0.925, 1.057) | 0.748 |
| WBC |  | 1.019(0.884, 1.175) | 0.797 |
| Serum potassium |  | 0.157(0.043, 0.573) | **0.005** |
| Serum Na |  | 0.876(0.740, 1.037) | 0.123 |
| Urea N |  | 0.992(0.940, 1.046) | 0.765 |
| Blood Cr |  | 0.985(0.961, 1.009) | 0.221 |
| Blood sugar |  | 1.154(0.943, 1.413) | 0.163 |
| ALB |  | 0.933(0.860, 1.013) | 0.100 |
| TB |  | 1.005(0.998, 1.011) | 0.164 |
| electrocardiogram |  | 1.366(0.680, 2.747) | 0.381 |
| Malignancy extent |  | 1.167(0.933, 1.459) | 0.175 |
| Urine 24h after surgery |  | 0.999(0.999, 1.000) | 0.169 |
| **Variables not presented in previous scoring system (We added)** | | | |
| Sex | female | 1.000 |  |
|  | male | 1.293(0.502, 3.325) | 0.595 |
| HBV |  | 0.664(0.275, 1.605) | 0.363 |
| HCV |  | 2.9046E-9(0, NA) | 0.999 |
| ALT |  | 1.000(0.996, 1.004) | 0.889 |
| AST |  | 1.002(0.997, 1.006) | 0.417 |
| ALP |  | 1.002(0.999, 1.004) | 0.218 |
| γ-GT |  | 1.001(1.000, 1.003) | 0.135 |
| DBIL |  | 1.005(0.997, 1.013) | 0.183 |
| IBIL |  | 1.029(0.998, 1.061) | 0.068 |
| PTA |  | 0.989(0.961, 1.017) | 0.436 |
| PT |  | 1.210(0.816, 1.794) | 0.344 |
| APTT |  | 0.964(0.870, 1.067) | 0.475 |
| TT |  | 1.077(0.880, 1.319) | 0.472 |
| AFP |  | 1.000(0.998, 1.001) | 0.497 |
| Tumor/no tumor |  | 1.076(0.443, 2.612) | 0.872 |

**Note:** ASA = American society of anesthesiologists; Hb = hemoglobin; HCT = hematocrit; WBC = white blood cell; Cr = creatinine; ALB = albumin; TB = total bilirubin; HBV = hepatitis B virus; HCV = hepatitis C virus; ALT = alanine aminotransferase; AST = aspartate aminotransferase; ALP = alkaline phosphatase; γ-GT = gamma- glutamyl transpeptidase; DBIL = direct bilirubin; IBIL = indirect bilirubin; PTA = prothrombin activity; PT = prothrombin time; APTT = activated partial thromboplastin time; TT = thrombin time; AFP = alpha fetoprotein; OR = odd ratio; CI = confidence interval. NA = not available; Bolded P value means it was lower than 0.05.

**Table S2. Postoperative complication in groups with** different score

| Score | Complication | | Total |
| --- | --- | --- | --- |
|  | No | Yes |  |
| 0 | 65 (94.20%) | 4 (5.80%) | 69 |
| 1 | 39 (90.70%) | 4 (9.30%) | 43 |
| 2 | 20 (64.52%) | 11 (35.48%) | 31 |
| 3 | 0 (0) | 7 (100%) | 7 |
| 0-1 | 104 (92.86%) | 8 (7.14%) | 112 |
| 2-3 | 20 (52.63%) | 18 (47.37%) | 38 |

**Note**: There were significant differences in postoperative complication rates among groups with different scores (χ²=48.856, P<0.001). When divided by 1.5 point, the incidence of postoperative complication between group with 0-1 points and 2-3 points was significantly different (χ²=32.041, P<0.001).

**Table S3. Definition for scales of surgery and corresponding scores**

| Score | Scale of surgery |
| --- | --- |
| 2 | Moderate hepatectomy:  Segmentectomy  Non-anatomical resection  Left later sectionectomy  Right anterior sectionectomy  Right posterior sectionectomy  Other resection of 2 segments |
| 4 | Major hepatectomy:  Right hepatectomy  Right hepatectomy with resection of segment 1  Extended right hepatectomy  Extended right hepatectomy with resection of segment 1  Left hepatectomy  Left hepatectomy with resection of segment 1  Extended left hepatectomy  Extended left hepatectomy with resection of segment 1  Central bisectionectomy (segments 4, 5 and 8)  All the Moderate hepatectomy with hepaticojejunostomy or bowel resection or other organ operation |
| 8 | Major+ hepatectomy:  All the Major hepatectomy with hepaticojejunostomy or bowel resection or other organ operation |
